# Hot water immersion acutely increases postprandial glucose concentrations

**DOI:** 10.14814/phy2.14223

**Published:** 2019-10-22

**Authors:** Christof A. Leicht, Lewis J. James, Jane H. B. Briscoe, Sven P. Hoekstra

**Affiliations:** ^1^ School of Sport, Exercise, and Health Sciences Loughborough University Loughborough United Kingdom; ^2^ The Peter Harrison Centre for Disability Sport Loughborough University Loughborough United Kingdom

**Keywords:** Glycemic control, hot water therapy, hyperthermia, passive heating, temperature

## Abstract

Chronic hot water immersion (HWI) confers health benefits, including a reduction in fasting blood glucose concentration. Here we investigate acute glycemic control immediately after HWI. Ten participants (age: 25 ± 6 years, body mass: 84 ± 14 kg, height 1.85 ± 0.09 m) were immersed in water (39°C) to the neck (HWI) or sat at room temperature (CON) for 60 min. One hour afterward they underwent an oral glucose tolerance test (OGTT), with blood collected before and after HWI/CON and during the 2 h OGTT. Glucose incremental area under the curve (iAUC) during the OGTT was higher for HWI (HWI 233 ± 88, CON 156 ± 79 mmol·L^−1^·2 h, *P* = 0.02). Insulin iAUC did not differ between conditions (HWI 4309 ± 3660, CON 3893 ± 3031 mU·L^−1^·2 h, *P* = 0.32). Core temperature increased to 38.6 ± 0.2°C during HWI, but was similar between trials during the OGTT (HWI 37.0 ± 0.2, CON 36.9 ± 0.4°C, *P* = 0.34). Directly following HWI, plasma average adrenaline and growth hormone concentrations increased 2.7 and 10.7‐fold, respectively (*P* < 0.001). Plasma glucagon‐like peptide‐1, peptide YY, and acylated ghrelin concentrations were not different between trials during the OGTT (*P* > 0.11). In conclusion, HWI increased postprandial glucose concentration to an OGTT, which was accompanied by acute elevations of stress hormones following HWI. The altered glycemic control appears to be unrelated to changes in gut hormones during the OGTT.

## Introduction

Passively increasing body temperature, by using hot water immersion (HWI) (Brunt et al. 2016; Hoekstra et al. 2018) or sauna bathing (Imamura et al. 2001; Biro et al. 2003; Laukkanen et al. 2015), can reduce risk markers related to inflammation and cardiovascular health. Moreover, HWI can lower the traditional blood‐derived markers associated with Type II Diabetes, such as fasting concentrations of glucose, insulin, or glycosylated hemoglobin after as little as 2 to 3 weeks (Hooper 1999; Hoekstra et al. 2018). Chronic HWI interventions further show improvements in glucose tolerance in rats being fed a high‐fat diet (Gupte et al. 2009), and a normalization of glucose excursions in Vervet monkeys (Kavanagh et al. 2016). These chronic effects of HWI are similar to responses observed following exercise training (Kränkel et al. 2019). HWI therapy has hence been suggested to represent a potential strategy to improve metabolic health for those unable to exercise (Hoekstra et al. 2018), much like exercise training has been advocated to be a suitable strategy to treat and prevent diseases associated with impaired glycemic control, such as Type II Diabetes (American Diabetes Association 2018).

A number of acute HWI effects likely contribute to the improvements in glycemic control following HWI therapy: Temperature per se appears to play a central role, as glucose uptake is increased when muscle temperature rises (Koshinaka et al. 2013). Importantly, in vitro work, removing the influence of circulating hormones and autonomic innervation, suggests a causative role for temperature in this context (Koshinaka et al. 2013). Furthermore, other putative mechanisms associated with heating likely enhance the independent effects of temperature. Fugmann et al. (2003) point out the importance of elevated blood flow for muscle glucose uptake. As ~3–4‐fold increases in leg blood flow are observed during passive heat stress (Chiesa et al. 2016), this likely is an additional explanatory factor. Jurcovicová et al. (1980) and Tatár et al. (1985) report acute hyperthermia‐related increases in glucagon and growth hormone concentrations, while Leicht et al. (2015) and Hashizaki et al. (2018) observed acute increases in plasma adrenaline and IL‐6 concentrations following HWI, all of which may impact on glycemic control. It is further possible that HWI‐induced changes in visceral blood flow impact on the concentration of gut hormones. These are implicated in glucose metabolism by stimulating (glucagon‐like peptide 1, GLP‐1) or inhibiting (ghrelin) insulin secretion; ghrelin has further been shown to act directly on the anterior pituitary to protect against hypoglycemia (Sun et al. 2019).

Repeated acute changes in glycemic control in response to regular HWI sessions may help to explain the chronic reductions in the traditional risk markers for chronic disease following HWI therapy. However, current evidence is limited as to whether HWI indeed is potent enough to induce such acute changes to glycemic control. The few studies that did investigate postprandial glycemic responses following HWI in humans have methodological limitations that make it difficult to draw firm conclusions. Some report results from small participant numbers and any conclusions regarding the glucose response might therefore be flawed due to insufficient statistical power (Jurcovicová et al. 1980). Others did not collect data in a resting control condition (Faulkner et al. 2017) failing to isolate the effect of HWI per se. Given the scarcity and limitations of HWI studies that investigated glycemic control, the primary aim of the present study was hence to establish whether an acute bout of HWI would impact on glucose concentrations during a subsequent OGTT.

## Materials and Methods

### Ethical approval

All procedures performed were in accordance with the ethical standards of the institutional committee (approval number R18‐P062) and with the 1964 Helsinki Declaration and its later amendments. Informed consent was obtained in writing from all individual participants included in the study.

### Participants

Twelve complete data sets from male participants were collected. Given the differential response to OGTT across the glucose tolerance spectrum (Knudsen et al. 2014), two participants were excluded from further analysis. Their 120 min OGTT glucose concentration in the control trial (CON; 7.4 and 7.9 mmol L^−1^, respectively) was elevated by more than 3 standard deviations from the rest of the group. These values are also in the range of the definition for impaired glucose tolerance (>7.8 mmol L^−1^) (Yudkin and Montori 2014). Ten participants were hence included for analysis (age: 25 ± 6 years; body mass: 84 ± 14 kg; height 1.85 ± 0.09 m, body fat: 14 ± 3%; peak oxygen uptake: 52 ± 10 mL kg^−1^ min^−1^).

### Experimental design

In the preliminary trial, participants were weighed to the nearest 0.1 kg (seca 770, Seca, Hamburg, Germany), and skinfold thickness was assessed at four sites (biceps, triceps, subscapular, and suprailiac) for the estimation of body fat percentage (Durnin and Womersley 1974). Participants' peak oxygen uptake was assessed with a ramp exercise test to exhaustion (start load: 20 W; ramp: 30 W min^−1^) on a cycle ergometer (Lode Excalibur Sport, Groningen, NL), using Douglas bags and a gas analyser (Servomex 1440, Servomex, Crowborough, UK).

Two main trials were conducted in a counterbalanced order. Participants arrived at 08:30 am after a 12 h overnight fast, having ingested a temperature monitor pill (HQInc, Palmetto FL) at 10 pm the previous night. On arrival, a cannula was inserted into a superficial vein of the forearm; patency of the cannula was maintained by flushing 10 mL of saline (0.9% NaCl) after each blood sample.

Following a 30 min rest, a resting expired gas sample and body mass were measured. In the HWI trial, participants were immersed to the neck (sternoclavicular notch) in a sitting position in hot water for 60 min. Water temperature was kept constant at 39.2 ± 0.2°C, measured continuously at the top and bottom of the tank (Squirrel, Grant Instruments, Shepreth, United Kingdom). Drinking water was given ad libitum during immersion and up to 15 min following immersion. Sweat loss between pre‐ and postimmersion was determined by body mass change and water intake. The procedures for CON were identical except participants rested on a chair at room temperature (23.2 ± 0.2°C, 46 ± 8% relative humidity) for 60 min instead of the water immersion, wearing shorts and a T‐shirt.

Following both interventions, participants sat at room temperature (23.5 ± 1.5°C, 49 ± 7% relative humidity) in shorts and T‐shirt and were allowed to do nonstrenuous tasks such as reading or watching television. Sixty minutes after the intervention period an OGTT was performed: 75 g of glucose (from dextrose monohydrate; Myprotein, Northwich, UK) dissolved in 300 mL of water was ingested, with participants remaining seated for the subsequent 120 min.

### Data collection

After removing the first 2 mL, blood samples were collected at pre‐ and postintervention, and at regular intervals during the OGTT: At 0 (just before ingestion of the glucose drink), and 15, 30, 45, 60, 90, and 120 min following ingestion. At all times of blood collection, an expired gas sample was obtained; additionally, expired gas samples were obtained at 15‐min intervals during HWI and CON. Metabolic rate in kJ h^−1^ was calculated using the equation 0.251·((3.914·${\dot{V}O}_{2}$) + (1.106·${\dot{V}\text{CO}}_{2}$)) (Weir 1949). Subjective ratings of hunger and fullness were reported on 100 mm visual analogue scales every 30 min, as described previously (Flint et al. 2000).

### Analytical methods

Blood was separated into three fractions. (1) Blood glucose concentration was directly measured from whole blood using a Biosen C‐line glucose analyser (Biosen, Barleben, Germany). (2) For all plasma analytes apart from acylated ghrelin, blood was collected into sterile K_3_EDTA containers, immediately centrifuged (2360 g, 10 min, 4°C; Allegra X‐22R Indianapolis) and the resulting plasma was stored at −80°C until analysis. (3) For the determination of acylated ghrelin, 2.7 mL whole blood was collected into K_3_EDTA containers pretreated with a 27 *μ*L solution containing potassium phosphate‐buffered saline, p‐hydroxymercuribenzoic acid, and NaOH (Clayton et al. 2016). Samples were then centrifuged at 2360*g* for 10 min after which 1 mL of the resulting plasma was mixed with 100 *μ*L of 1 mol L^−1^ HCl. Acidified samples were centrifuged for a further 5 min at 11,000*g* (AccuSpin Micro 17, Fisher Scientific, Hampton, US) before being stored at −80°C until analysis.

Analyte plasma concentrations were determined using commercially available enzyme‐linked immunosorbent assay (ELISA) kits, coefficients of variation (CV) determined through duplicates analysis are indicated for each: *adrenaline* (CV 5.3%), Tecan UK Ltd, Reading, UK*; growth hormone* (CV 9.3%), R&D, Abingdon, UK; *insulin* (CV 3.4%*),* Mercodia, Uppsala, Sweden; and gut hormones *glucagon‐like peptide‐1* (*GLP‐1,* CV 5.0%*)*, *peptide YY* (*PYY,* CV 7.7%*),* Merck Millipore, Watford, UK*; ghrelin* (CV 1.8%*),* Bioquote Ltd, York, UK; according to the manufacturers' instructions using a microplate reader (Varioskan Flash, ThermoScientific, Waltham). Adrenaline and growth hormone were analyzed at pre‐ and postintervention, ghrelin, GLP‐1, and PYY additionally during the OGTT (at 0, 30, 60, and 120 min), and glucose and insulin at all sampled time points. All samples from the same participant were analyzed on the same microplate.

Incremental areas under the curve (iAUC) for glucose and insulin during the OGTT were calculated using the trapezoidal rule using time point zero as baseline.

### Power calculation, data processing, and statistical analyses

A power calculation was performed using GPower 3.1.9.2 (Kiel, Germany) based on data presented by Jurcovicová et al. (1980). We calculated that *N* = 10 would be required to detect a significant difference in peak glucose concentration between conditions with an *α* of 0.05 and a power of 80%.

The SPSS 23 statistical package (SPSS Inc., Chicago IL) was used for all statistical analyses. Normality was checked with the Shapiro–Wilk test statistic. Means and standard deviations were computed for all variables; data violating normality assumptions were converted using logarithmic transformations, which resulted in normal distributions for all converted data sets. Two‐way (condition by time) repeated measures analyses of variance (ANOVA) with Huynh–Feldt correction where assumptions of sphericity were violated were performed for the pre/post comparisons of the intervention, and for the 2 h period of the OGTT. Where significant, interaction effects were further investigated using Bonferroni‐corrected paired‐sample student *T*‐tests. Paired‐sample student *T*‐tests were also used to compare iAUC between conditions. Statistical significance was accepted at *P* < 0.05.

## Results

### Glucose and insulin

**Figure 1:**
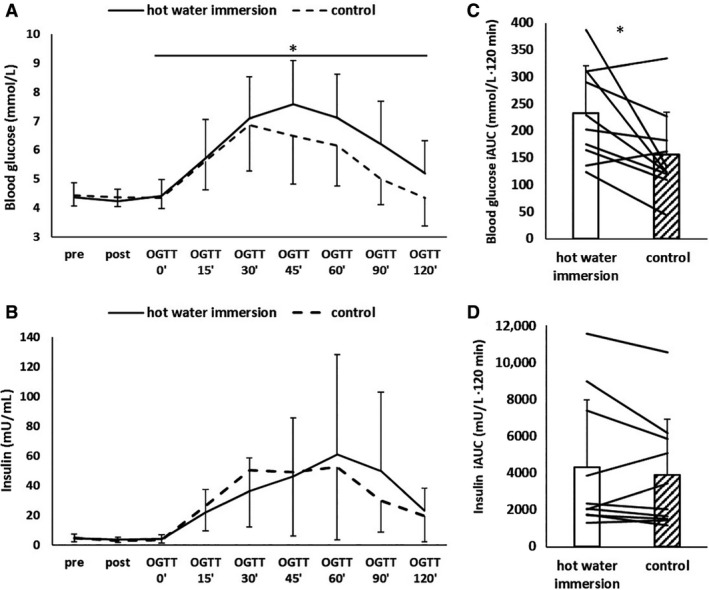
Blood glucose and plasma insulin concentrations in response to an oral glucose tolerance test (OGTT) after hot water immersion or control. (A and B) time course (means and standard deviations); (C and D) Incremental area under the curve (iAUC) for the whole duration of the OGTT for glucose and insulin (bars and whiskers: means and standard deviations, lines: individual responses). Main effect of time observed for both glucose and insulin; *difference between conditions, at *P* < 0.05.

HWI resulted in higher blood glucose concentrations during the OGTT than CON (*P* = 0.02, Fig. 1). As a result, glucose iAUC during the OGTT was higher for HWI (HWI 233 ± 88, CON 156 ± 79 mmol L^−1^·120 min, *P* = 0.02, Fig. 1). This was due to a higher iAUC in HWI in the second hour of the OGTT (*P* = 0.05); iAUC in the first hour of the OGTT did not differ between conditions (*P* = 0.73). Insulin plasma concentrations during the OGTT (*P* = 0.31, Fig. 1) and insulin iAUC (HWI 4305 ± 3655, CON 3889 ± 3029 mU L^−1^·120 min, *P* = 0.45) were not different between conditions.

Blood glucose and plasma insulin concentrations were reduced from pre to post (*P* < 0.05); however, the pattern of the decline was not affected by HWI (both Time × Condition effects *P* = 0.41, Fig. 1).

### Stress and gut hormones

**Figure 2:**
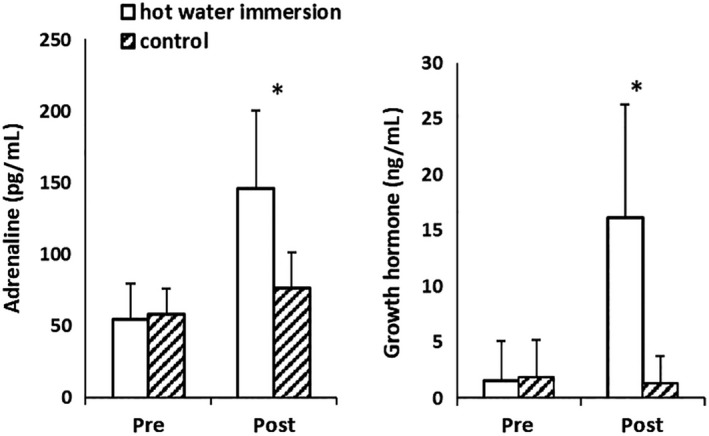
Plasma adrenaline and growth hormone concentrations in response to hot water immersion or control (means and standard deviations). *Difference between conditions, at *P* < 0.05.

Time × condition interactions were found for both plasma adrenaline and growth hormone concentrations (*P* < 0.001, Fig. 2): At preintervention, plasma adrenaline and growth hormone concentrations did not differ between conditions (both *P* > 0.35), but were both greater postintervention (both *P* < 0.001; Fig. 2).

**Figure 3:**
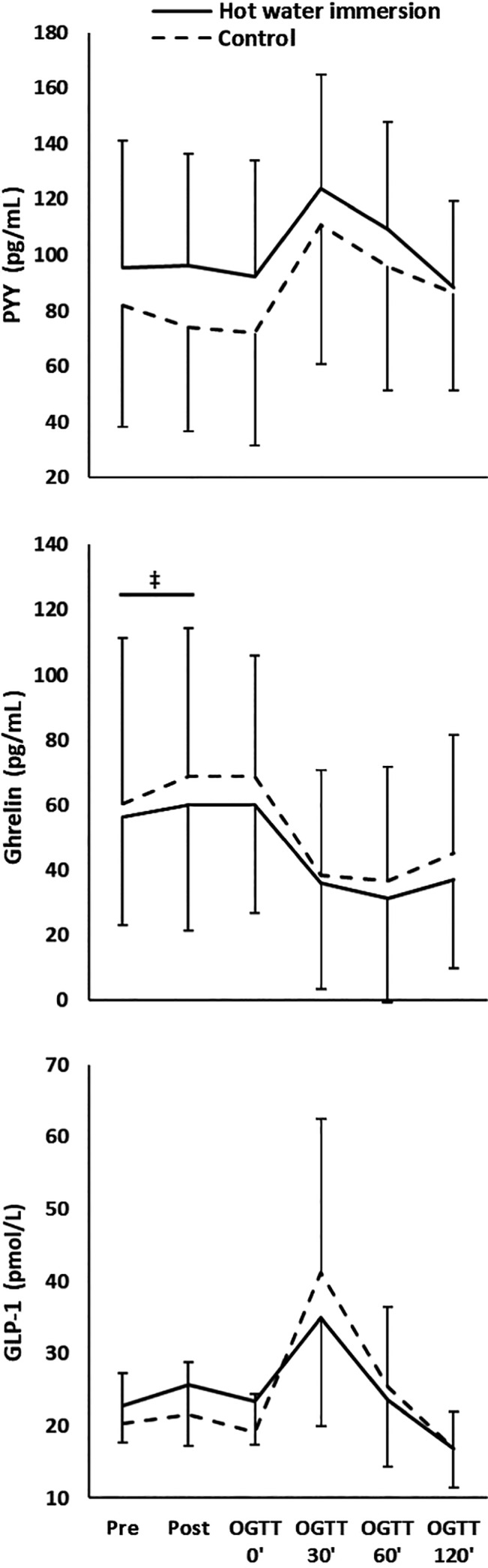
Gut hormone plasma concentrations in response to hot water immersion or control (means and standard deviations). OGTT, oral glucose tolerance test. Main effect of time observed for all hormones; ^‡^time × condition interaction, at *P* < 0.05.

Main effects of time during the OGTT were found for all gut hormones (*P* < 0.005), with an increase in the plasma concentrations of PYY and GLP‐1, and a decrease in acylated ghrelin concentration during the OGTT. However, plasma PYY, GLP‐1, and acylated ghrelin concentrations did not differ between conditions throughout the OGTT (*P* > 0.11, Fig. 3).

There was no acute effect of HWI on GLP‐1 and PYY (no time × condition interactions for the pre/post comparison; *P* > 0.35). However, a time × condition interaction for acylated ghrelin (*P* = 0.02) indicated a blunted ghrelin response from pre‐ to post‐HWI: during CON, mean plasma acylated ghrelin concentration increased by +8.6 ± 9.7 pg mL^−1^, (*P* = 0.01; mean change 14%), during HWI, the change was + 3.9 ± 15.3 pg mL^−1^ (*P* = 0.67, mean change 7%).

Perceived hunger did not differ between conditions during the OGTT (*P* = 0.23, Table 1). From pre to post, a main effect of time indicated an increase in perceived hunger (*P* = 0.006), but this was not different between conditions (Time × Condition *P* = 0.59, Table 2). Perceived fullness did not differ between conditions during the OGTT (*P* = 0.23, Table 1). A larger increase in fullness was found from pre to post during HWI (Time × Condition *P* = 0.01, Table 2).

**Table 1:** Physiological data, perceived hunger, and fullness during the oral glucose tolerance test following hot water immersion (HWI) or control (CON).

Parameter	Condition	Oral glucose tolerance test (min)						
		0	15	30	45	60	90	120
Core temperature (°C)‡	HWI	37.2 ± 0.3	37.1 ± 0.3	37.0 ± 0.3	37.0 ± 0.3	37.0 ± 0.3	37.0 ± 0.2	37.0 ± 0.2
	CON	36.8 ± 0.4	36.8 ± 0.5	36.8 ± 0.4	36.8 ± 0.4	36.9 ± 0.3	36.9 ± 0.4	37.0 ± 0.4
Respiratory exchange ratio†	HWI	0.77 ± 0.10	0.75 ± 0.09	0.77 ± 0.09	0.84 ± 0.11	0.82 ± 0.08	0.86 ± 0.11	0.85 ± 0.07
	CON	0.77 ± 0.04	0.72 ± 0.07	0.77 ± 0.09	0.80 ± 0.08	0.83 ± 0.07	0.84 ± 0.05	0.83 ± 0.08
Resting metabolic rate (kJ·h^−1^)†	HWI	374 ± 51	392 ± 63	399 ± 69	416 ± 65	410 ± 66	411 ± 69	397 ± 53
	CON	388 ± 61	393 ± 71	415 ± 71	413 ± 81	396 ± 61	408 ± 60	389 ± 47
Perceived hunger (0–10)	HWI	8.4 ± 1.6		7.6 ± 1.8		7.5 ± 2.4	7.4 ± 2.3	7.5 ± 2.2
	CON	8.6 ± 1.3		7.8 ± 1.9		7.8 ± 1.9	8.1 ± 1.2	8.2 ± 1.6
Perceived fullness (0–10)	HWI	1.5 ± 1.5		1.9 ± 1.4		1.9 ± 1.6	2.4 ± 2.1	2.4 ± 2.4
	CON	1.3 ± 1.4		2.0 ± 1.7		2.5 ± 1.9	1.7 ± 1.3	1.6 ± 1.5

Data are means and standard deviations.

^†^ Main effect of time.

^‡^ Time × condition interaction (*P* < 0.05).

**Table 2:** Physiological data, perceived hunger, and fullness during hot water immersion (HWI) or control (CON). Data are means and standard deviations.

Parameter	Condition	Hot water immersion/Control (min)				
		Pre	15	30	45	60 (post)
Core temperature (°C)*, †, ‡	HWI	36.9 ± 0.2	37.5 ± 0.3	38.0 ± 0.2	38.3 ± 0.2	38.6 ± 0.2
	CON	36.9 ± 0.3	37.0 ± 0.3	37.0 ± 0.3	36.9 ± 0.3	36.9 ± 0.4
Respiratory exchange ratio*†	HWI	0.77 ± 0.11	0.86 ± 0.10	0.87 ± 0.10	0.88 ± 0.15	0.89 ± 0.20
	CON	0.80 ± 0.11	0.83 ± 0.08	0.82 ± 0.07	0.79 ± 0.05	0.79 ± 0.06
Resting metabolic rate (kJ·h^−1^)^*†‡^	HWI	346 ± 43	468 ± 76	515 ± 92	519 ± 95	538 ± 105
	CON	360 ± 90	419 ± 111	427 ± 131	395 ± 75	394 ± 82
Perceived hunger (0–10)†	HWI	6.4 ± 1.6		6.8 ± 1.6		7.9 ± 1.5
	CON	6.9 ± 1.7		7.7 ± 1.6		8.0 ± 1.3
Perceived fullness (0–10)*, †, ‡	HWI	2.1 ± 1.3		3.4 ± 2.0		2.4 ± 1.5
	CON	2.3 ± 1.3		2.0 ± 1.5		1.7 ± 1.6

* Main effect of condition.

^†^ Main effect of time.

^‡^ Time × condition interaction (*P* < 0.05).

### Thermoregulatory and metabolic responses

Resting core temperature was 36.9 ± 0.2°C and 36.9 ± 0.3°C for HWI and CON, respectively (*P* = 0.79). After 60 min of HWI, this increased to 38.6 ± 0.2°C (*P* < 0.001) but did not change during CON (36.9 ± 0.4 °C; *P* = 0.71, Table 2). While core temperature at the start of the OGTT tended to be greater after HWI (HWI 37.2 ± 0.3°C, CON 36.8 ± 0.4°C, *P* = 0.09), this trend disappeared at 15 min into the OGTT (HWI 37.1 ± 0.3°C, CON 36.9 ± 0.3°C, *P* = 0.44). Consequently, core temperature throughout the OGTT did not differ between conditions (HWI 37.0 ± 0.2°C, CON 36.9 ± 0.4°C, *P* = 0.18, Table 1).

During immersion, main effects of time (*P* < 0.05) and time × condition interactions were observed for RER and metabolic rate (*P* < 0.05), with higher values towards the end of the HWI trial (Table 2). No time × condition interactions were found for either measures during the OGTT (*P* > 0.35, Table 1). During HWI, sweat loss was 1.4 ± 0.2 L and water ingested was 0.8 ± 0.6 L; sweat loss was 0.1 ± 0.1 L and water ingested was 0.3 ± 0.3 L during CON. This resulted in body mass changes of −0.79 ± 0.55% during HWI and +0.16 ± 0.27% during CON.

## Discussion

The main findings of this study are as follows: (1) HWI leads to elevated postprandial blood glucose concentrations during a subsequent OGTT; (2) the gut hormone response and perceived hunger and fullness during the OGTT are unaffected by HWI; and (3) acute elevations of plasma adrenaline and growth hormone concentrations, and in perceived fullness, are found immediately after HWI.

This is the first study to report elevated postprandial glucose concentrations during an OGTT subsequent to HWI when compared to a resting control condition. While not all exercise studies investigating OGTT responses postexercise show this change in circulating glucose (Bonen et al. 1998), a similar increase in postprandial glucose has been found after 60 min of submaximal constant load exercise in humans with normal glycemic control (Rose et al. 2001; Knudsen et al. 2014). It has been argued that residual effects of elevated stress hormones may be responsible for altered glycemic control (Knudsen et al. 2014). For example, beta‐adrenergic stimulation of epithelial cells by adrenaline increases glucose absorption in sheep (Aschenbach et al. 2002), and it was hence argued that this might increase orally ingested exogenous glucose appearance (Knudsen et al. 2014). Growth hormone can impair insulin sensitivity (Yuen et al. 2013), and both catecholamines and growth hormone can increase hepatic glucose output (Dufour et al. 2009; Yuen et al. 2013). Catecholamines can further suppress insulin‐mediated glucose transport into skeletal muscle (Hunt and Ivy 2002). If these stress hormones do help orchestrate the glycemic response as is suggested for exercise, they may play a similar role in HWI. However, while the plasma adrenaline and growth hormone concentrations were acutely elevated following HWI in the present study, it is important to point out that the half‐life of these hormones is rather short. Adrenaline concentrations return to baseline within ~30 min of recovery from exercise (Weltman et al. 2000) or HWI (Jimenez et al. 2007; Whitham et al. 2007; Laing et al. 2008); growth hormone concentrations return to baseline within ~60–90 min following exercise (Weltman et al. 2000) or HWI (Jurcovicová et al. 1980). As the differences in glucose concentration between conditions in the present study were found in the second half of the OGTT (120–180 min post‐HWI), the influence of catecholamines and growth hormone on glycemic control is likely to be indirect, by inducing processes with longer lasting effects. In addition to the effect on stress hormones, temperature can independently increase tissue glucose uptake (Koshinaka et al. 2013). However, the differences in glucose concentration between conditions occurred in the second half of the OGTT, when core temperature did not differ between conditions. The rest period between HWI and the OGTT, lasting 60 min, was designed to allow core temperature to return to resting levels during this crucial observation period. The *direct* stimulatory effect of temperature (Koshinaka et al. 2013), together with any *direct* stimulatory effect of stress hormones (Aschenbach et al. 2002; Hunt and Ivy 2002) on increased appearance of glucose, hence do not explain the altered glucose kinetics during the OGTT. In addition, insulin is unlikely to be an explanatory factor for the difference in glucose iAUC as insulin concentration and insulin iAUC during the OGTT did not differ between conditions, which is in line with earlier exercise (Rose et al. 2001; Knudsen et al. 2014) and HWI (Jurcovicová et al. 1980) studies. Interestingly though, higher elevations in glucose concentration without any changes in the insulin response do imply a temporary reduction in insulin sensitivity following HWI.

The investigation that most closely resembles the present study was published by Jurcovicová et al. (1980), exploring OGTT responses following HWI, and employing a control condition of immersion in thermoneutral water. In contrast to the present results, they reported no apparent effect of HWI on responses to a glucose challenge. However, visual inspection of their glucose data indicates a trend of delayed response following HWI – in line with the present results. It must further be noted that their research was limited to a small number of participants (*N* = 6), consisting of a subset of growth hormone responders from a larger participant pool. We therefore conclude that our data do not contradict the findings of this early study but contend that this previous study was simply underpowered to detect any differences in postprandial glucose responses. We further argue that we have employed a more ecologically valid approach by conducting a control trial resting at room temperature, rather than in thermoneutral water. Indeed, it is possible that differences in hydrostatic pressure between conditions might contribute to the observed effects.

We also note that the increased postprandial glucose response found in the present study is in line with studies where, in contrast to the present study, core temperature was elevated *during* the OGTT (Tatár et al. 1985; Dumke et al. 2015; Kimball et al. 2018). This implies that the elevated temperature per se might not have been a crucial parameter to induce the changes in glycemic control in these studies. Further, HWI resulted in an increased sweat rate compared to CON while fluid intake was greater during HWI. This produced a very low level of dehydration (<1% body mass) in the HWI trial that was not apparent in the CON trial. However, given dehydration of 1–2% body mass does not appear to influence postprandial glucose responses during an OGTT (Carroll et al. 2019), this is unlikely to contribute to the observed effects.

In line with the present study, swimming (i.e., exercise performed in water) does not alter the postprandial acylated ghrelin concentrations after exercise (King et al. 2011). Further, HWI did not impact on plasma GLP‐1 and PYY concentrations during the OGTT. This is a noteworthy finding, as an increased GLP‐1 response to an OGTT is predictive of chronic reductions in fasting glucose (Koopman et al. 2005). The present results hence imply that the chronic reductions in blood glucose following HWI therapy (Hooper 1999; Hoekstra et al. 2018) are not the result of any acute HWI‐induced changes to the acute GLP‐1 response. The only differential gut hormone response was observed directly following HWI, when the increase in acylated ghrelin concentration was blunted when compared with CON. Even though this blunted response in the present study was modest, the results are again in line with the swimming exercise study of King et al. (2011). We conclude that while a small acute variation in the gut hormone response was found immediately following HWI, it did not result in a differential gut hormone response during the OGTT and is unlikely to impact energy intake or eating behavior.

Subjective perceptions of fullness are likely to be temporarily influenced by hydrostatic pressure, which was indeed the case when participants were immersed in the present study. This is in line with the increases in perceptions of fullness reported with swimming (King et al. 2011). It is possible that the water compression of the abdomen stimulated mechanoreceptors involved in perception of fullness (Carmagnola et al. 2005). Furthermore, it is possible that the increased fluid intake during HWI increased this perception (Corney et al. 2016). However, it is important to note that this did not change perception of hunger between conditions, consistent with Carmagnola et al. (2005) who found perception of hunger to be unaffected by changes in fullness. In conclusion, hunger perception and the gut hormone response did not differ between conditions, but HWI increased the resting metabolic rate. This provides some rationale for HWI therapy to affect energy balance, which may go some way to explain the reductions in body mass following thermal therapy (Hooper 1999; Imamura et al. 2001).

This study sought to broaden our understanding as to why chronic HWI interventions result in improvements of risk markers associated with glucose metabolism (Hooper 1999; Gupte et al. 2009; Kavanagh et al. 2016; Hoekstra et al. 2018). Future investigations should focus on the relevance of acute elevations in postprandial glucose concentrations, also reported following exercise (Rose et al. 2001; Knudsen et al. 2014), and whether they indeed cause improvements in fasting measures of glucose metabolism.

## Conclusions

A more pronounced increase in blood glucose concentration was observed during the OGTT subsequent to HWI compared with seated rest at room temperature, despite no difference in core temperature or plasma insulin concentration during the OGTT. This change in glycemic control might be explained by the residual effect of stress hormones, which were acutely elevated following HWI. The gut hormone response during the OGTT was unaffected by HWI, therefore unlikely to explain the difference in the observed glucose response between conditions. Future research should determine whether these acute changes in glycemic control are causally linked to the chronic reductions in fasting blood glucose concentration following HWI therapy reported elsewhere.

## Conflict of Interest

The authors declare no conflict of interest.

## References

[phy214223-bib-0001] American Diabetes Association . 2018 5. Prevention or Delay of Type 2 Diabetes: Standards of Medical Care in Diabetes—2018. Diabetes Care 41(Suppl. 1):S51–S54. 10.2337/dc18-S005. 29222376

[phy214223-bib-0002] Aschenbach, J. R. , T. Borau , and G. Gäbel . 2002 Glucose uptake via SGLT‐1 Is stimulated by β2‐adrenoceptors in the ruminal epithelium of sheep. J. Nutr. 132:1254–1257. 10.1093/jn/132.6.1254. 12042442

[phy214223-bib-0003] Biro, S. , A. Masuda , T. Kihara , and C. Tei . 2003 Clinical implications of thermal therapy in lifestyle‐related diseases. Exp. Biol. Med. (Maywood, NJ) 228:1245–1249. 10.1177/153537020322801023 14610268

[phy214223-bib-0004] Bonen, A. , M. Ball‐Burnett , and C. Russel . 1998 Glucose tolerance is improved after low‐ and high‐intensity exercise in middle‐age men and women. Can. J. Appl. Physiol. 23:583–593. 9822794 10.1139/h98-033

[phy214223-bib-0005] Brunt, V. E. , M. J. Howard , M. A. Francisco , B. R. Ely , and C. T. Minson . 2016 Passive heat therapy improves endothelial function, arterial stiffness, and blood pressure in sedentary humans. J. Physiol. 594:5329–5342. 10.1113/JP272453. 27270841 PMC5023696

[phy214223-bib-0006] Carmagnola, S. , P. Cantu , and R. Penagini . 2005 Mechanoreceptors of the proximal stomach and perception of gastric distension. Am. J. Gastroenterol. 100:1704–1710. 10.1111/j.1572-0241.2005.41350.x. 16086705

[phy214223-bib-0007] Carroll, H. A. , I. Templeman , Y.‐C. Chen , R. M. Edinburgh , E. K. Burch , J. T. Jewitt , et al. 2019 Effect of acute hypohydration on glycemic regulation in healthy adults: a randomized crossover trial. J. Appl. Physiol. 126:422–430. 10.1152/japplphysiol.00771.2018. 30496706 PMC6397405

[phy214223-bib-0008] Chiesa, S. T. , S. J. Trangmar , and J. González‐Alonso . 2016 Temperature and blood flow distribution in the human leg during passive heat stress. J. Appl. Physiol. 120:1047–1058. 10.1152/japplphysiol.00965.2015. 26823344 PMC4894946

[phy214223-bib-0009] Clayton, D. J. , D. J. Stensel , and L. J. James . 2016 Effect of breakfast omission on subjective appetite, metabolism, acylated ghrelin and GLP‐17‐36 during rest and exercise. Nutrition 32:179–185. 10.1016/j.nut.2015.06.013. 26421384

[phy214223-bib-0010] Corney, R. A. , C. Sunderland , and L. J. James . 2016 Immediate pre‐meal water ingestion decreases voluntary food intake in lean young males. Eur. J. Nutr. 55:815–819. 10.1007/s00394-015-0903-4. 25893719

[phy214223-bib-0011] Dufour, S. , V. Lebon , G. I. Shulman , and K. F. Petersen . 2009 Regulation of net hepatic glycogenolysis and gluconeogenesis by epinephrine in humans. Am. J. Physiol.‐Endocrinol. Metab. 297:E231–E235. 10.1152/ajpendo.00222.2009. 19458062 PMC2711660

[phy214223-bib-0012] Dumke, C. L. , D. R. Slivka , J. S. Cuddy , W. S. Hailes , S. M. Rose , and B. C. Ruby . 2015 The effect of environmental temperature on glucose and insulin after an oral glucose tolerance test in healthy young men. Wild. Environ. Med. 26:335–342. 10.1016/j.wem.2015.03.002. 25937547

[phy214223-bib-0013] Durnin, J. V. , and J. Womersley . 1974 Body fat assessed from total body density and its estimation from skinfold thickness: measurements on 481 men and women aged from 16 to 72 years. Br. J. Nutr. 32:77–97. 4843734 10.1079/bjn19740060

[phy214223-bib-0014] Faulkner, S. H. , S. Jackson , G. Fatania , and C. A. Leicht . 2017 The effect of passive heating on heat shock protein 70 and interleukin‐6: A possible treatment tool for metabolic diseases? Temperature 4:292–304. 10.1080/23328940.2017.1288688. PMC5605168 28944271

[phy214223-bib-0015] Flint, A. , A. Raben , J. E. Blundell , and A. Astrup . 2000 Reproducibility, power and validity of visual analogue scales in assessment of appetite sensations in single test meal studies. Int. J. Obes. Relat. Metab. Disord. 24:38–48. 10702749 10.1038/sj.ijo.0801083

[phy214223-bib-0016] Fugmann, A. , M. Sarabi , B. Karlström , C. Berne , H. Lithell , and L. Lind . 2003 Blood flow is an important determinant of forearm glucose uptake following a mixed meal. Acta Diabetol. 40:113–117. 10.1007/s00592-003-0098-7. 14605966

[phy214223-bib-0017] Gupte, A. A. , G. L. Bomhoff , R. H. Swerdlow , and P. C. Geiger . 2009 Heat treatment improves glucose tolerance and prevents skeletal muscle insulin resistance in rats fed a high‐fat diet. Diabetes 58:567–578. 10.2337/db08-1070. 19073766 PMC2646055

[phy214223-bib-0018] Hashizaki, T. , Y. Nishimura , K. Teramura , Y. Umemoto , M. Shibasaki , C. A. Leicht , et al. 2018 Differences in serum IL‐6 response after 1 °C rise in core body temperature in individuals with spinal cord injury and cervical spinal cord injury during local heat stress. Int. J. Hyperth. 35:541–547. 10.1080/02656736.2018.1511838. 30303416

[phy214223-bib-0019] Hoekstra, S. P. , N. C. Bishop , S. H. Faulkner , S. J. Bailey , and C. A. Leicht . 2018 The acute and chronic effects of hot water immersion on inflammation and metabolism in sedentary, overweight adults. J. Appl. Physiol. 125:2008–2018. 10.1152/japplphysiol.00407.2018. 30335579

[phy214223-bib-0020] Hooper, P. L. 1999 Hot‐tub therapy for type 2 diabetes mellitus. N. Engl. J. Med. 341:924–925. 10.1056/NEJM199909163411216. 10498473

[phy214223-bib-0021] Hunt, D. G. , and J. L. Ivy . 2002 Epinephrine inhibits insulin‐stimulated muscle glucose transport. J. Appl. Physiol. 93:1638–1643. 10.1152/japplphysiol.00445.2002. 12381748

[phy214223-bib-0022] Imamura, M. , S. Biro , T. Kihara , S. Yoshifuku , K. Takasaki , Y. Otsuji , et al. 2001 Repeated thermal therapy improves impaired vascular endothelial function in patients with coronary risk factors. J. Am. Coll. Cardiol. 38:1083–1088. 11583886 10.1016/s0735-1097(01)01467-x

[phy214223-bib-0023] Jimenez, C. , B. Melin , G. Savourey , J. C. Launay , A. Alonso , and J. Mathieu . 2007 Effects of passive hyperthermia versus exercise‐induced hyperthermia on immune responses: hormonal implications. Eur. Cytokine Netw. 18:154–161. ecn.2007.0101. 17823084 10.1684/ecn.2007.0101

[phy214223-bib-0024] Jurcovicová, J. , M. Vigas , M. Palát , D. Jezová , and I. Klimes . 1980 Effect of endogenous GH secretion during hyperthermic bath on glucose metabolism and insulin release in man. Endocrinol. Exp. 14:221–226. 7002532

[phy214223-bib-0025] Kavanagh, K. , A. T. Davis , K. A. Jenkins , and D. M. Flynn . 2016 Effects of heated hydrotherapy on muscle HSP70 and glucose metabolism in old and young vervet monkeys. Cell Stress Chaperones 21:717–725. 10.1007/s12192-016-0699-z. 27188431 PMC4908005

[phy214223-bib-0026] Kimball, A. L. , P. M. McCue , M. A. Petrie , and R. K. Shields . 2018 Whole body heat exposure modulates acute glucose metabolism. Int. J. Hyperth. 35:644–651. 10.1080/02656736.2018.1516303. 30303421

[phy214223-bib-0027] King, J. A. , L. K. Wasse , and D. J. Stensel . 2011 The acute effects of swimming on appetite, food intake, and plasma acylated ghrelin. J. Obes. 2011:1–8. 10.1155/2011/351628. PMC2952805 20953411

[phy214223-bib-0028] Knudsen, S. H. , K. Karstoft , B. K. Pedersen , G. van Hall , and T. P. J. Solomon . 2014 The immediate effects of a single bout of aerobic exercise on oral glucose tolerance across the glucose tolerance continuum. Physiol. Rep. 2:e12114 10.14814/phy2.12114. 25168869 PMC4246585

[phy214223-bib-0029] Koopman, R. , R. J. F. Manders , A. H. G. Zorenc , G. B. J. Hul , H. Kuipers , H. A. Keizer , et al. 2005 A single session of resistance exercise enhances insulin sensitivity for at least 24 h in healthy men. Eur. J. Appl. Physiol. 94:180–187. 10.1007/s00421-004-1307-y. 15761746

[phy214223-bib-0030] Koshinaka, K. , E. Kawamoto , N. Abe , K. Toshinai , M. Nakazato , and K. Kawanaka . 2013 Elevation of muscle temperature stimulates muscle glucose uptake in vivo and in vitro. J. Physiol. Sci. 63:409–418. 10.1007/s12576-013-0278-3. 23836025 PMC10718043

[phy214223-bib-0031] Kränkel, N. , M. Bahls , E. M. Van Craenenbroeck , V. Adams , L. Serratosa , E. E. Solberg , et al. 2019 Exercise training to reduce cardiovascular risk in patients with metabolic syndrome and type 2 diabetes mellitus: How does it work? Eur. J. Prev. Cardiol. 26:701–708. 10.1177/2047487318805158. 30317879

[phy214223-bib-0032] Laing, S. J. , A. R. Jackson , R. Walters , E. Lloyd‐Jones , M. Whitham , N. Maassen , et al. 2008 Human blood neutrophil responses to prolonged exercise with and without a thermal clamp. J. Appl. Physiol. 104:20–26. 10.1152/japplphysiol.00792.2007. 17901240

[phy214223-bib-0033] Laukkanen, T. , H. Khan , F. Zaccardi , J. A. Laukkanen . 2015 Association between sauna bathing and fatal cardiovascular and all‐cause mortality events. JAMA Intern. Med. 175:542–548. 10.1001/jamainternmed.2014.8187. 25705824

[phy214223-bib-0034] Leicht, C. A. , K. Kouda , Y. Umemoto , M. Banno , T. Kinoshita , T. Moriki , et al. 2015 Hot water immersion induces an acute cytokine response in cervical spinal cord injury. Eur. J. Appl. Physiol. 115:2243–2252. 10.1007/s00421-015-3206-9. 26105530

[phy214223-bib-0036] Rose, A. J. , K. Howlett , D. S. King , and M. Hargreaves . 2001 Effect of prior exercise on glucose metabolism in trained men. Am. J. Physiol.‐Endocrinol. Metab. 281:E766–E771. 10.1152/ajpendo.2001.281.4.E766. 11551853

[phy214223-bib-0037] Sun, E. W. L. , A. M. Martin , R. L. Young , and D. J. Keating . 2019 The regulation of peripheral metabolism by gut‐derived hormones. Front. Endocrinol. 9:754 10.3389/fendo.2018.00754. PMC6328484 30662430

[phy214223-bib-0038] Tatár, P. , M. Vigas , J. Jurcovicová , D. Jezová , V. Strec , and M. Palát . 1985 Impaired glucose utilization in man during acute exposure to environmental heat. Endocrinol. Exp. 19:277–281. 3910408

[phy214223-bib-0039] Weir, J. 1949 New methods for calculating metabolic rate with special reference to protein metabolism. J. Physiol. 109:1–9. 15394301 10.1113/jphysiol.1949.sp004363 PMC1392602

[phy214223-bib-0040] Weltman, A. , C. J. Pritzlaff , L. Wideman , J. Y. Weltman , J. L. Blumer , R. D. Abbott , et al. 2000 Exercise‐dependent growth hormone release is linked to markers of heightened central adrenergic outflow. J. Appl. Physiol. 89:629–635. 10.1152/jappl.2000.89.2.629. 10926647

[phy214223-bib-0041] Whitham, M. , S. J. Laing , A. Jackson , N. Maassen , and N. P. Walsh . 2007 Effect of exercise with and without a thermal clamp on the plasma heat shock protein 72 response. J. Appl. Physiol. 103:1251–1256. 10.1152/japplphysiol.00484.2007. 17673560

[phy214223-bib-0042] Yudkin, J. S. , and V. M. Montori . 2014 The epidemic of pre‐diabetes: the medicine and the politics. BMJ (Clinical Research Ed) 349:18–20. 10.1136/bmj.g4485. PMC4707710 25028385

[phy214223-bib-0043] Yuen, K. C. J. , L. E. Chong , and M. C. Riddle . 2013 Influence of glucocorticoids and growth hormone on insulin sensitivity in humans. Diabet. Med. 30:651–663. 10.1111/dme.12184. 23510125

